# Zoonotic malaria requires new policy approaches to malaria elimination

**DOI:** 10.1038/s41467-023-41546-6

**Published:** 2023-09-16

**Authors:** Kimberly M. Fornace, Chris J. Drakeley, Kim A. Lindblade, Jenarun Jelip, Kamruddin Ahmed

**Affiliations:** 1 Saw Swee Hock School of Public Health, National University of Singapore, Singapore, Singapore; 2 School of Biodiversity, One Health and Veterinary Medicine, University of Glasgow, Glasgow, UK; 3 Faculty of Infectious and Tropical Diseases, London School of Hygiene and Tropical Medicine, London, UK; 4 PATH, Geneva, Switzerland; 5 Ministry of Health, Kota Kinabalu, Malaysia; 6 Borneo Medical and Health Research Centre, Universiti Malaysia Sabah, Kota Kinabalu, Malaysia; 7 Department of Pathology and Microbiology, Faculty of Medicine and Health Sciences, Universiti Malaysia Sabah, Kota Kinabalu, Malaysia; 8 Research Center for Global and Local Infectious Diseases, Oita University, Oita, Japan

**Keywords:** Malaria, Policy, Epidemiology

## Abstract

WHO guidelines for classification of malaria elimination in a country require that the risk of human infection from zoonotic, as well as nonzoonotic, malaria parasites is negligible. In this Comment, the authors discuss the implications of this policy for countries, such as Malaysia, with no recent reported nonzoonotic cases but ongoing zoonotic transmission.

## Teaser

Increasing numbers of human zoonotic malaria cases are reported globally. Current malaria control measures cannot eliminate transmission from wildlife reservoirs, leaving many countries with no pathway to malaria elimination certification. New policies are needed to redefine elimination goals and certification.

## Defining malaria elimination

A world free of malaria is the declared vision of the global health community^1^. Elimination of malaria at a country level, defined as the interruption of local malaria transmission, is a critical step towards realising this vision. Since the 1960s, the World Health Organization (WHO) has certified countries that achieved elimination, requiring demonstration of zero indigenous malaria cases for three years and a programme for prevention of re-establishment of transmission^2^. In 2020, the WHO E-2020 initiative contributed to the achievement of global elimination milestones with 10 more countries achieving elimination; current targets aim to eliminate from 35 countries by 2030^1, 3^.

Malaria elimination certification officially recognises substantial investment and provides a measurable achievement of immense public health importance by national programmes. WHO malaria elimination certification also signals redirection of malaria surveillance and control measures towards preventing re-establishment of malaria transmission^4^. Notably, the lack of indigenous malaria transmission does not imply there is no malaria risk in the country but rather that malaria risks are likely to come from imported cases. This necessitates prioritising areas with high malariogenic potential and refining surveillance systems that can detect, respond and contain emerging risks^5^. Although specific measures vary by epidemiological context, this shifts resources away from control measures aiming to reduce transmission in local human populations and towards mitigating vectorial capacity and managing malaria importations.

## The impact of emerging zoonotic malaria on elimination

Previous WHO guidelines defined malaria elimination as interruption of transmission of the four nonzoonotic malaria parasites (*Plasmodium falciparum*, *P. vivax*, *P. ovale*, and *P. malariae*) and specifically excluded the zoonotic malaria *P. knowlesi*^6^. However, in 2022, revised certification guidelines additionally require that risk from other *Plasmodium* species be considered no more than negligible^5,7^. While “negligible risk” has not been officially defined, initial discussions suggested this may amount to ten or fewer zoonotic malaria cases per year^7^. This change had the most immediate implications for certification of malaria elimination in Malaysia; although Malaysia has not reported any indigenous cases of the four nonzoonotic malaria parasites since 2018, it reports thousands of human cases of the zoonotic malaria *P. knowlesi* annually^8^. Carried by macaques and transmitted by the *Anopheles* leucosphyrus group of mosquitoes, increasing evidence suggests deforestation is driving *P. knowlesi* spillover from wildlife to humans^9^.

The rapid rise in *P. knowlesi* cases in humans in Malaysia raises the question of whether *P. knowlesi* is evolving to become another nonzoonotically transmitted malaria parasite. However, while human-mosquito-human *P. knowlesi* transmission is possible, our review of existing evidence suggests this is highly unlikely^10^. We used model-based inference methods to analyse over 25,000 *P. knowlesi* cases in Malaysia reported from 2012 – 2020 and found no evidence of sustained nonzoonotic *P. knowlesi* transmission. Additionally, our analysis identified pronounced differences in transmission patterns of *P. knowlesi* from those of the nonzoonotic malaria parasites *P. falciparum* and *P. vivax*^8^. Standard control measures, such as insecticide-treated nets and rapid detection and treatment of human cases, have successfully controlled nonzoonotic malaria but have had no appreciable impact on zoonotic malaria case incidence in Malaysia, likely due to these differences in transmission and the presence of a wildlife reservoir.

While Malaysia currently reports the highest number of zoonotic malaria cases globally and is at a critical stage for elimination certification, zoonotic malaria is a global issue. Human cases of *P. knowlesi* and other zoonotic simian malaria parasites have been reported across Southeast Asia in many countries targeting malaria elimination by 2030^11^. For example, Thailand aims to eliminate malaria by 2024 but reported over 200 *P. knowlesi* cases in the first half of 2023^12,13^. In South America, human cases of the zoonotic malarias *P. simium* and *P. brasilianum* have been reported in previously malaria-free regions^14^. In contrast to *P. knowlesi*, these simian malaria parasites can cause sustained nonzoonotic transmission in human populations^15^. In West Africa, limited evidence suggests nonzoonotic parasites may also spill back into wildlife populations and later spillover into human populations^16^. The complexity of the zoonotic malaria transmission dynamics, where wildlife can act as a reservoir for malaria parasites infecting humans or a source of emerging malaria parasites, poses substantial barriers to malaria elimination certification as currently defined by WHO.

## The need for new guidance and systems to address zoonotic malaria

What is the way forward for malaria elimination where there is a zoonotic reservoir? While there is yet no evidence of sustained nonzoonotic transmission of *P. knowlesi*, similarly to imported cases, there is a need to ensure that health systems are capable of identifying and treating cases to prevent severe morbidity or mortality and reduce the chances of onward transmission, even if the risk of onward transmission to other humans is minimal to nonexistent. For zoonotic malaria, control guidance can be made more robust by identifying areas with high spillover risks, alerting the population and travellers and ensuring resources (e.g., vector control measures) are targeted to these areas and populations. Additionally, while not a current requirement, surveillance systems should collect the epidemiological and genetic data required to monitor potential changes in transmission patterns. While logistical and financial barriers still exist, the implementation of genomic surveillance for SARS-COV-2 demonstrates how genetic analysis can be incorporated into routine surveillance to monitor pathogen populations^17^. Similarly, mathematical modelling tools using routinely collected data, such as date of symptom onset and address, can be used to estimate reproductive numbers and assess likelihood of nonzoonotic transmission^8^.

Although it is tempting to view Malaysia’s zoonotic malaria burden and pathway to malaria elimination certification as an isolated issue, zoonotic malaria is likely to be a major barrier to certification in many countries aiming for elimination by 2030. Increasing numbers of *P. knowlesi* cases have been identified across Southeast Asia, including in Indonesia and Thailand, and zoonotic malaria is reported in multiple countries in South America^10^. Zoonotic malaria risks are strongly associated with deforestation and increased overlap between humans, non-human primates and mosquito vectors at forest edges^18^. With increased rates of global environmental change, zoonotic malaria is likely to be a continued public health threat in the future^19^. These ecological impacts on transmission dynamics may be compounded by decreasing immunity to other malaria species following successful malaria control programmes or influxes of susceptible populations into areas experiencing land use change.

There is a critical need both to manage this disease risk and prevent zoonotic malaria from making malaria elimination certification unobtainable for many countries and derailing global elimination efforts. The Dahlem criteria, establishing priorities for diseases that could be eradicated or eliminated, recognise that zoonotic diseases cannot be eliminated without highly effective interventions or extermination of the animal reservoir^20^. Correspondingly, WHO recognises three levels of disease control and elimination: eradication, elimination of transmission and elimination as a public health problem^20^. Criteria for certification of elimination would likely be better served by recognising the different surveillance and control requirements for managing nonzoonotic and zoonotic malaria risks and developing separate certification pathways to recognise countries’ achievements. While elimination of transmission is possible for the four main nonzoonotic malaria parasites, the elimination of zoonotic malaria appears feasible only in the context of a public health problem. Similarly to assessing malaria importation, this would require surveillance systems to collect the data required to discriminate between transmission within local human populations and introductions from other sources, such as wildlife.

Zoonotic malaria can cause severe and fatal malaria in people and remains a public health concern that should not be neglected^21^. Recent pandemics highlight the continued threat of pathogen spillover from wildlife. As our research illustrates, these spillover events likely happen frequently and can cause a substantial public health burden even when they do not lead to transmission within human populations. With limited interventions for diseases driven by spillover, increased investment in research is required to develop novel control measures to manage infection in wildlife reservoirs to decrease human disease risks. As unprecedented levels of global environmental change increase opportunities for pathogen exchange between species, there is an urgent need for policies to manage zoonotic disease risks while supporting wider disease control and elimination efforts.

## Policy recommendations

- New approaches to certification of malaria elimination are needed for countries reporting only cases of zoonotic malaria and having eliminated nonzoonotic malaria species. This should include separate elimination certification pathways by transmission modes. While elimination of transmission is possible for nonzoonotic malaria parasites, it is unlikely to be feasible for zoonotic malaria.
- Without interventions to control zoonotic malaria in the animal reservoir, zoonotic malaria in humans should be a target for elimination as a public health problem. Specific criteria should be established to validate that zoonotic malaria is no longer a public health problem.
- Malaria surveillance systems need to incorporate methods of collecting and analysing genetic and epidemiological data to evaluate evidence of zoonotic and nonzoonotic transmission and detect changes in transmission pathways that may signal adaptation to transmission within human populations only.
- Increased research and investment are required to identify control measures to reduce spillover risks and achieve elimination goals in countries where zoonotic malaria species circulate in wildlife.
